# Melting phase relations in Fe–Si–H at high pressure and implications for Earth’s inner core crystallization

**DOI:** 10.1038/s41598-022-14106-z

**Published:** 2022-06-15

**Authors:** Koutaro Hikosaka, Shoh Tagawa, Kei Hirose, Yoshiyuki Okuda, Kenta Oka, Koichiro Umemoto, Yasuo Ohishi

**Affiliations:** 1 Department of Earth and Planetary Science, The University of Tokyo, Bunkyo, Tokyo 113-0033 Japan; 2 Earth-Life Science Institute, Tokyo Institute of Technology, Meguro, Tokyo 152-8550 Japan; 3 Japan Synchrotron Radiation Research Institute, SPring-8, Sayo, Hyogo 679-5198 Japan

**Keywords:** Solid Earth sciences, Core processes, Geochemistry, Mineralogy, Petrology

## Abstract

Hydrogen could be an important light element in planetary cores, but its effect on phase diagrams of iron alloys is not well known because the solubility of H in Fe is minimal at ambient pressure and high-pressure experiments on H-bearing systems have been challenging. Considering that silicon can be another major light element in planetary cores, here we performed melting experiments on the Fe–Si–H system at ~ 50 GPa and obtained the ternary liquidus phase relations and the solid/liquid partition coefficient, *D* of Si and H based on *in-situ* high-pressure X-ray diffraction measurements and *ex-situ* chemical and textural characterizations on recovered samples. Liquid crystallized hexagonal close-packed (hcp) (Fe_0.93_Si_0.07_)H_0.25_, which explains the observed density and velocities of the Earth’s solid inner core. The relatively high *D*_Si_ = 0.94(4) and *D*_H_ = 0.70(12) suggest that in addition to Si and H, the liquid outer core includes other light elements such as O, which is least partitioned into solid Fe and can thus explain the density difference between the outer and inner core. H and O, as well as Si, are likely to be major core light elements, supporting the sequestration of a large amount of water in the Earth’s core.

## Introduction

Since Birch^1^ reported the density deficit and velocity excess of the Earth’s outer core with respect to pure iron (Fe), light elements in the core have long been explored but still remain controversial^2^. Recent planet formation theories suggested that a large amount of water could have been delivered to the growing Earth^3–5^. The chemical reaction of water with Fe metals in a magma ocean led to the incorporation of hydrogen (H) along with silicon (Si) and oxygen (O) into the core^6–9^. While O is least partitioned into solid Fe and should therefore be negligible in the inner core^10–12^, both Si and H could be present in both the outer and inner core. Indeed, measurements of the density and sound velocity of solid Fe and Fe alloys supported that Si^13,14^ and H^15,16^ are important impurity elements in the inner core. Recent theoretical calculations^17^ suggested that the Earth’s solid inner core is an hcp Fe_60_Si_4_H_1–15_ alloy, depending on the temperature at the inner core boundary (ICB), *T*_ICB_ = 5500–6500 K.

The liquidus phase diagrams of Fe alloys and the solid metal-liquid metal partitioning of these light elements are of great importance to constrain possible ranges of the outer and inner core compositions^2^. Those of Fe–Si alloys have been repeatedly examined at high pressures up to inner core conditions^18–22^. In contrast, the phase relations of H-bearing Fe alloys are little known^23–25^. This is because (1) only a negligible amount of H can be present in liquid/solid Fe at 1 bar and therefore H escapes from Fe lattice during decompression^6,9,26^ and (2) high-pressure experiments on H-bearing systems often involve technical problems such as the failure of a diamond-anvil. Recent neutron diffraction measurements are able to reveal the position and abundance of H atoms in solid Fe alloys under high pressure but so far limited to less than 12 GPa^27,28^. The properties of hcp Fe–Si–H ternary alloys, including the simultaneous solubilities of Si and H, have not been examined at high pressure and high temperature (*P–T*)^29^.

In this study, we have investigated the liquidus relations and the solid/liquid partitioning of Si and H in the Fe–Si–H ternary system at ~ 50 GPa based on melting experiments in a laser-heated diamond-anvil cell (DAC). The results demonstrated that an hcp Fe–Si–H alloy, similar in composition to those predicted for the inner core solid^17^, crystallized from liquid with *D*_Si_ (solid/liquid) = 0.94(4) and *D*_H_ = 0.70(12) on a weight basis. The relatively high *D*_Si_ and *D*_H_ require the inclusion of other light elements in the outer core such as O, in order to account for a density contrast between the outer and inner core. We explored the possible liquid core composition and found that H and O, in addition to Si, are likely to be important light elements in the Earth’s core.

## Methods

### High P–T experiments

Laser-heated DAC techniques were used to generate high *P–T* conditions. The starting materials were ~ 10 µm thick Fe–Si foils containing 4.0 and 6.5 wt% Si. The culet size of the diamond anvils used was either 200 µm or 300 µm depending on the hydrogen source, and the sample chamber was prepared by drilling a hole with a diameter of ~ 80 µm or ~ 100 µm, respectively, in a rhenium gasket pre-indented to ~ 25 µm thickness. Hydrogen was introduced to the system by sandwiching the Fe–Si sample between C_n_H_2n+2_ paraffin layers (run #4) or cryogenically loading liquid hydrogen into the sample chamber^30^ (runs #1–#3, #5). In the latter case, an NaCl ring prepared with a focused ion beam (FIB) was employed inside the rhenium gasket as an H_2_ insulator and a pressure medium. In addition, the culet of the diamond anvils was coated with a thin layer of Ti by sputtering to avoid anvil failure^31^. After cooling the DAC to 20 K with the sample chamber open, the chamber was flooded with liquid H_2_^30^. The sample was then sealed by weak compression and brought back to room temperature. The presence of hydrogen in the sample chamber was confirmed by Raman spectroscopy. Subsequently, it was further compressed to ~ 20 GPa and weakly annealed at < 1000 K to prompt hydrogenation of the alloy. The hydrogen content was different in each run because of differences in temperature and duration of the thermal annealing and in the quantity of H_2_ directly in contact with Fe–Si alloys. In order to remove excess hydrogen, the DAC was then again cooled to ~ 90 K using liquid N_2_ and decompressed back to 1 atm, so that any hydrogen in excess was released from the sample chamber while preventing the decomposition of hydrogenated iron^32^.

Heating was conducted at the beamline BL10XU, SPring-8 synchrotron radiation facility using a couple of 100 W single-mode Yb fiber lasers (YLR-100, IPG Photonics) with flat-top beam shaping optics^9^. The samples were heated to melting temperatures for a limited duration of ~ 3 s in an attempt to avoid temperature fluctuation. This time scale has been shown to be long enough to reach chemical equilibrium due to the short length scale of DAC samples; in previous DAC melting experiments, liquid and coexisting solid compositions did not change after 1 s in the Fe–S system^33^. We obtained sample temperature profiles using spectroradiometry and estimated the temperature at the liquid–solid boundary (liquidus temperature) in combination with a sample cross section^12,22^ (Table 1).

**Table 1:** Experimental conditions and results.

Run #	*P* (GPa)	*T* (K)		Phase	Fe wt%	Si wt%	H wt%	*x*^a^	C wt%	O wt%
1	56(3)	2100(210)	Before	hcp	96.0	4.0	0.19(2)	0.10	–	–
			After	hcp	97.5(9)	3.7(1)	0.47(4)	0.25	0.0(1)	0.1(1)
				Liquid (fcc)	97.9(7)	4.0(1)	0.67(5)	0.35	0.0(1)	0.1(1)
2	57(3)	2150(110)	Before	hcp	96.0	4.0	0.58(5)	0.31	–	–
			After	hcp	96.2(8)	4.3(3)	0.30(2)	0.16	0.2(1)	0.1(1)
				Liquid (hcp)	92.6(20)	5.9(1)	0.56(4)	0.30	0.0(1)	0.5(3)
3	61(3)	2350(400)	Before	hcp	96.0	4.0	1.54(12)	0.81	–	–
			After	fcc	99.7(12)	0.8(2)	1.59(13)	0.87	0.3(2)	0.3(2)
				Liquid (hcp)	93.2(13)	5.8(2)	0.55(4)	0.29	0.4(3)	0.7(4)
4	48(2)	2100(210)	Before	hcp	93.5	6.5	–	–	–	–
			After	fcc	98.9(17)	0.3(2)	1.76(14)	0.98	0.1(1)	0.2(1)
				Liquid (dhcp)	94.2(10)	5.6(2)	1.84(15)	0.97	0.6(1)	0.5(2)
				B2	87.5(5)	13.4(2)	0.82	0.4	0.4(1)	0.2(1)
5	49(2)	2450(120)	Before	hcp	96.0	4.0	1.86(15)	0.99	–	–
			After	fcc	100.4(5)	0.6(1)	1.78(14)	0.98	0.1(1)	0.1(1)
				Liquid (fcc)	91.9(4)	7.1(1)	1.79(14)	0.93	0.5(1)	0.7(2)

Phases found before and after heating are given with their chemical compositions. The metal starting materials were Fe + 6.5 wt%Si in run #4 and Fe + 4.0 wt%Si in other runs. Numbers in parentheses indicate errors in the last digits. ^a^*x* in (Fe,Si)H_*x*_*.*

### Sample analyses

**Figure 1:**
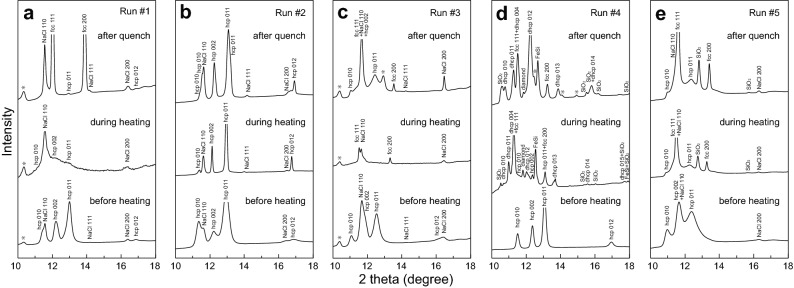
Sample XRD patterns collected at high pressures in (**a**–**e**) runs #1 to #5 before, during and after heating (melting). Asterisks indicate unknown peaks. See text for details.

*In-situ* angle-dispersive X-ray diffraction (XRD) measurements were conducted before, during and after heating (melting) using an X-ray beam with an energy of ~ 30 keV^9^ (Figs. 1, S1 and S2). The beam was focused on a sample using compound refractive lenses and collimated so that the full-width at half maximum was 6 μm. The 2D diffraction patterns were collected on a flat panel detector (Perkin Elmer), and integrated to 1D patterns using the IPAnalyzer software and analyzed with the PDIndexer software^34^. Pressure was estimated on the basis of the unit-cell volume of CsCl-type NaCl^35^ considering its temperature following Ref.^36^, except for run #4 where it was obtained from the Raman shift of diamond^37^ and corrected for a contribution of thermal pressure^12,38^. The overall uncertainty should be ± 5% (Table 1).

**Figure 2:**
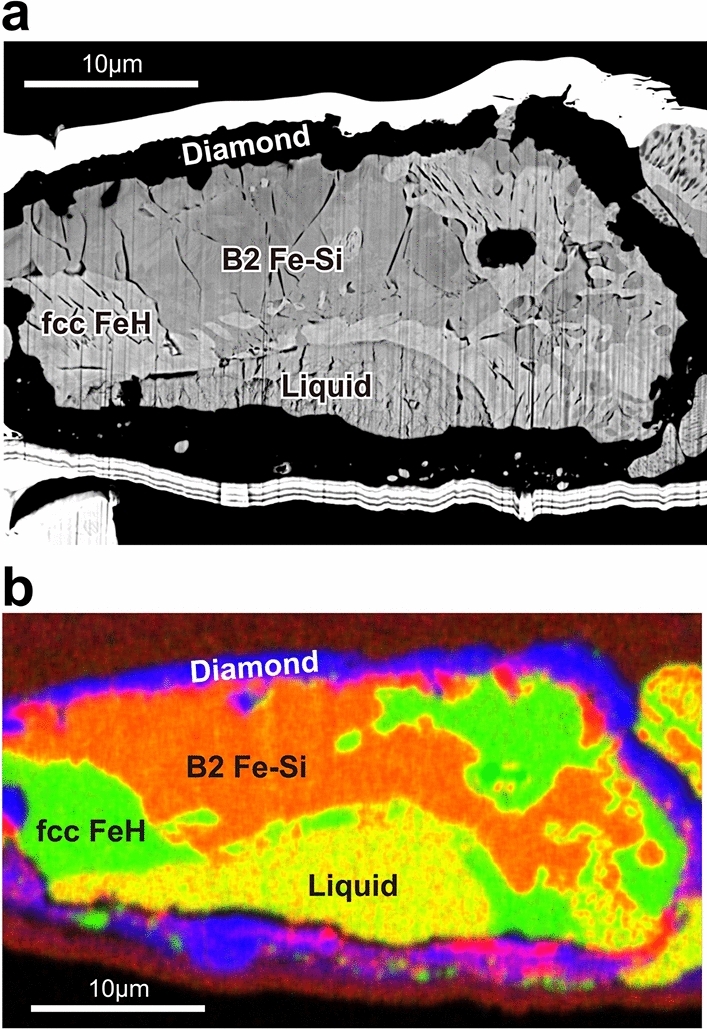
Coexisting Fe–Si–H liquid and solid fcc FeH and B2 Fe–Si phases at 48 GPa and 2100 K in run #4 using Fe–Si and C_n_H_2n+2_ paraffin as starting materials. (**a**) Back-scattered electron image and (**b**) combined X-ray elemental map of Fe (green) + Si (red) + C (blue). Bubbles and cracks in (**a**) indicate that hydrogen was present in liquid and solids at high pressure and escaped during decompression.

Textural and chemical characterizations were carried out on all the samples after decompression and recovery (Figs. 2 and S3). A Ga-ion FIB (VersaTM 3D DualBeamTM, FEI) was used to mill the sample parallel to the compression axis and prepare a cross section of the laser-heated portion. The sample was then examined by a field-emission (FE)-type scanning electron microscope (SEM) and energy dispersive X-ray spectrometry (EDS). Furthermore, a field-emission-type electron probe micro-analyzer (FE-EPMA, JEOL JXA-8530F) was used for quantitative chemical analyses with an accelerating voltage of 12 kV and a current of 15 nA. We used Fe, Si, Fe–0.84 wt%C and Fe_3_C as standards, and LIF (Fe), PETH (Si), LDE2H (C) and LDE1 (O) as analyzing crystals. Carbon (C) concentration was quantified from a calibration curve obtained by a C-free copper mesh, Fe–0.84 wt%C (JSS066-6, the Japan Iron and Steel Federation) and Fe_3_C.

Since hydrogen escapes from iron lattice at low pressure (less than ~ 3 GPa) and room temperature^6,9,39^, we determined the H content *x* of (Fe,Si)H_*x*_ based on the volume expansion of the Fe–Si crystal lattice at room temperature^23^ (Fig. S4);

1 $$x = \frac{{V_{{\left( {{\text{Fe{-}Si}}} \right){\text{H}}_{x} }} - V_{{{\text{Fe{-}Si}}}} }}{{\Delta V_{{\text{H}}} }}$$

where *V* is lattice volume and Δ*V*_H_ is the volume increase caused per H atom^40^. The reference lattice volume of Fe–Si at identical high pressure was estimated from those of pure Fe^41,42^ and Fe–6.5 wt%Si^20^. The H abundance in liquid was estimated from the volume of face-centered cubic (fcc) or hcp crystals formed upon quenching temperature. The use of other equation of state for an Fe–Si alloy^43^ increases the *x* values only by 0.01–0.04. The effect of possible minor inclusion of carbon in these crystals was not considered. The validity of such estimation has been demonstrated in Refs.^9^ and^44^; thermal annealing of such quench crystals changed their volume to a minor extent, indicating that their H concentration represents that of liquid. The EPMA analyses and the H contents are summarized in Table 1. The error in H concentration should be ± 8%, which is derived mainly from uncertainty in Δ*V*_H_^9,28^.

## Results

### Liquidus phase relations in Fe–Si–H

**Figure 3:**
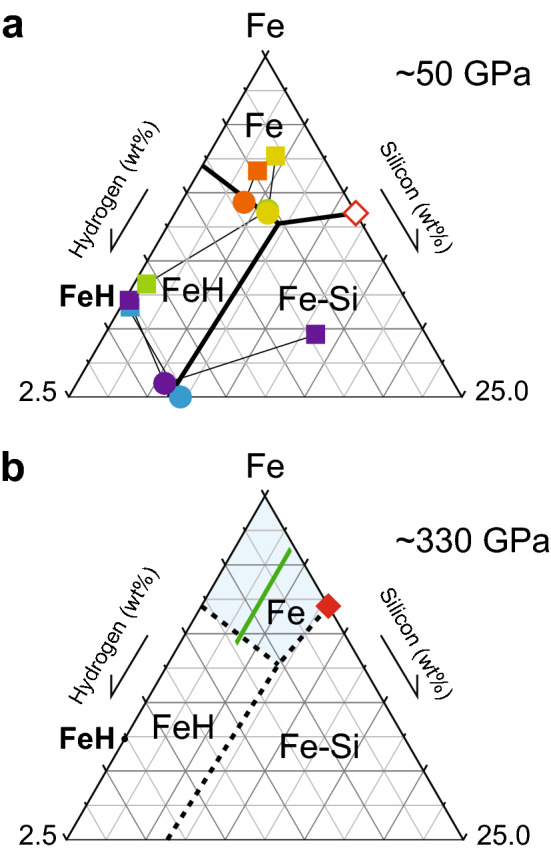
Liquidus phase relations in Fe–Si–H showing the liquidus fields of hcp Fe, fcc FeH and B2 Fe–Si at (**a**) ~ 50 GPa and (**b**) ~ 330 GPa. The compositions of liquids (circles) and coexisting solid phases (squares) obtained in the present experiments are plotted in (**a**); orange, run #1; yellow, run #2; green, run #3; purple, run #4; blue, run #5. Open and closed red diamonds in (**a**) and (**b**) show the Fe–FeSi eutectic liquid composition at respective pressure^22^. The liquid core compositions (green belt), calculated with *D*_Si_ and *D*_H_ considering their uncertainties from the proposed inner core solid compositions^17^, are mostly within the liquidus field of (Si, H)-bearing hcp Fe (blue area) at 330 GPa (**b**). Note that stochiometric FeSi includes 33.5 wt% Si.

We performed five separate experiments in the Fe–Si–H ternary system at ~ 50 GPa, in which C and O concentrations in liquids were less than 0.6 and 0.7 wt%, respectively (Table 1). The coexisting liquid and solid (liquidus phase) compositions are plotted in Fig. 3a, which constrains the liquidus fields of hcp Fe, fcc FeH and B2 Fe–Si.

In the first two runs, liquids coexisted with (Si, H)-bearing hcp Fe. In run #1, the XRD pattern showed hydrogenated hcp Fe + 4 wt%Si together with the NaCl pressure medium before heating at 56 GPa (Figs. 1a, S2 and S3a). During heating to 2100 K, we observed diffuse scattering signal from liquid around two-theta angle of 12°, coexisting with weak reflections from the hcp phase (liquidus phase). Upon quenching to 300 K, the fcc phase appeared. Combined with microprobe analyses of the cross section of the recovered sample, these observations indicated that liquid Fe–4.0 wt%Si–0.67 wt%H coexisted with hcp Fe–3.7 wt%Si–0.47 wt%H during heating. Similarly in run #2 performed at 57 GPa, the XRD pattern collected at 2150 K included the peaks from the hcp phase and the NaCl pressure medium (Figs. 1b and S3b). The diffuse signal was not clear, which is consistent with the microprobe observation of a small melt pocket at one side of the recovered sample surface. Liquid Fe–5.9 wt%Si–0.56 wt%H coexisted with hcp Fe–4.3 wt%Si–0.30 wt%H at high *P–T* in this experiment. It is, however, uncertain that this solid composition represents that of the liquidus phase, because the small liquid pool suggests that the coexisting liquidus phase was volumetrically even smaller and the determination of its chemical composition was difficult.

On the other hand, liquids coexisted with fcc (nearly) stoichiometric FeH in other runs. In run #3, hcp (Fe,Si)H_0.81_ was found before heating at 61 GPa (Figs. 1c and S3c). Upon heating to 2350 K, the peaks from the hcp phase were lost, and alternatively those of fcc appeared together with the diffuse scattering signal from liquid. The XRD pattern obtained after quenching temperature and the EPMA analyses on the recovered sample showed that liquid Fe–5.8 wt%Si–0.55 wt%H crystallized Si-free fcc stoichiometric FeH during melting. In run #4, an H-rich liquid (Fe,Si)H_0.97_ coexisted with both fcc stoichiometric FeH and B2 Fe–13.4 wt%Si–H (Figs. 1d and 2). The unit-cell volume of B2 Fe–Si found in this experiment (18.5 Å^3^ at 42.6 GPa and 300 K) was 4% smaller than that of H-free B2 Fe + 13.4 wt%Si (19.2 Å^3^) at the equivalent condition that was estimated from previous volume measurements of B2 Fe–Si^45^ (Fig. S4). It is difficult to estimate its H content from the smaller unit-cell volume of B2; alternatively, we approximated it from the relative proportions of bubbles and cracks between the B2 and neighboring stoichiometric FeH in the cross section of this sample (Fig. 2a). It gives an approximate amount of H in the B2 phase to be (Fe,Si)H_0.4_. The recent experiments by Ref.^46^ also reported a reduction in the volume of B2 stoichiometric FeSi upon hydrogenation and argued that it can be caused by the substitutional incorporation of H into the B2 structure. In run #5, we observed that H-rich liquid (Fe,Si)H, which was similar in composition to that in run #4, coexisted with fcc stoichiometric FeH (Figs. 1e and S3d).

Figure 3a illustrates the compositions of these liquids and coexisting solids (liquidus phase) plotted in the Fe–Si–H ternary diagram. The recent experiments^22^ reported the Fe–FeSi binary eutectic liquid composition to be Fe + 11.5 wt% Si at 50 GPa, which is helpful to obtain the ternary liquidus phase relations. While the liquids formed in runs #2 and #3 were similar to each other, they coexisted with hcp Fe and fcc FeH, respectively. It indicates that these two liquid compositions are close to the Fe + FeH cotectic line (showing liquids coexisting two solid phases). The liquid in run #1 coexisted with the hcp phase, which also constrains the location of this Fe + FeH cotectic line. The H-rich liquid found in run #4 crystallized both fcc FeH and B2 Fe–Si (Fig. 2) and is therefore on the FeH + Fe–Si cotectic line. It is consistent with the result of run #5.

These observations show each liquidus field (showing a compositional range of liquids that first crystallize a given solid phase) of Fe, FeH and Fe–Si at ~ 50 GPa (Fig. 3a). Considering that we often obtained liquids with compositions close to binary eutectic point or ternary cotectic lines in similar melting experiments on Fe alloy systems such as Fe–Fe_3_S^33^ and Fe–S–O^12^, the results of runs #1–#3 suggest the Fe–FeH binary eutectic composition to be around FeH_0.45_ at 50 GPa.

The liquidus temperatures (temperatures at solid/liquid boundary) found in runs #1–#3 were 2100–2350 K, which are much lower than the Fe–FeSi binary eutectic temperature of 2900 K at 50 GPa^19^, indicating a large effect of hydrogen to reduce melting temperature^24,44^. The ternary invariant point at which liquid coexists with three solid phases (Fe, FeH and Fe–Si) may be a peritectic (not eutectic) point at ~ 2400 K.

### Solid–liquid partitioning of Si & H

The solid/liquid partition coefficients of Si and H between (Si, H)-bearing hcp Fe and coexisting liquid were obtained in run #1, where strong diffuse signal from liquid was observed in the high-temperature XRD pattern (Fig. 1a); *D*_Si_ = 0.94(4) and *D*_H_ = 0.70(12) (weight basis) (Table 1). The *D*_Si_ = 0.94(4) is consistent with those previously observed in the H-free Fe–FeSi binary system at ambient and high pressures^18,21^.

Fcc stoichiometric FeH included the least amounts of Si when coexisting with Fe–Si–H liquids (Fig. 3a), suggesting that Si atoms do not substitute Fe when octahedral interstitial sites are fully occupied by H atoms^28^. On the other hand, the chemical composition of the B2 phase formed in run #4 was estimated to be (Fe_0.77_Si_0.23_)H_0.4_, which is enriched in both Si and H than hcp (Fe_0.93_Si_0.07_)H_0.25_ found in run #1. The *D*_H_ (B2/liquid) could be 0.45, certainly smaller than the *D*_H_ (hcp/liquid) = 0.70(12).

## Discussion

### Crystallization of hcp Fe–Si–H at Earth’s inner core

The solid inner core of our planet consists of hcp Fe containing some light elements; the inner core density deficit with respect to pure Fe has been estimated to be about 4%^41,47^. While the least amounts of O and C are incorporated into solid Fe in the inner core^10,12,22^, Si, H and S are known to form solid solution with Fe to some extent^18,19,23,33^ and likely present in the solid core. Nevertheless, interactions among Si, H and S atoms in hcp Fe could be strong and affect their simultaneous solubilities as well as solid–liquid partitioning^48,49^. Indeed, the liquid immiscibility, a typical consequence of the strong interaction, has been observed between Fe–H and Fe–S liquids to > 100 GPa^50^.

Recent ab initio simulations performed by Refs.^17^ and ^51^ emphasized the presence of C and/or H in the solid inner core, in order to account for not only the density but the low P- and S-wave velocities observed. As mentioned above, C is unlikely to be an important impurity element in the inner core because of its low *D*_C_ (solid-hcp/liquid) ~ 0.1^22^; otherwise the liquid core should be enriched in C, which is not compatible with its density and velocity observations^52,53^. Alternatively the inner core alloy may be H-bearing hcp Fe_60_Si_4_H_1–15_ when *T*_ICB_ ranges from 5500 to 6500 K (more H is necessary for lower *T*_ICB_)^17^.

The present high-pressure experiments demonstrated that liquid crystallized hcp (Fe_0.93_Si_0.07_)H_0.25_ (Fe_60_Si_4.5_H_16_), which is almost equivalent to the inner core solid proposed by Ref.^17^ when *T*_ICB_ is 5500 K. Although Ref.^29^ examined the compression behaviors of hcp (Fe_0.88_Si_0.12_)H_0.61–0.79_ at 300 K, this study first confirmed that the hcp Fe–Si–H alloy proposed for the inner core is stable to melting temperatures. While *D*_H_ (solid-Fe/liquid) has never been reported in the literature, *D*_Si_ = 0.94(4) obtained in this study is similar to those previously found in the Fe–Si system, indicating no remarkable dependence on H concentration in liquid^18,21^. It contrasts the large effects of sulfur (S)^48,49^ and C^22^, which remarkably enhance the solid/liquid *D*_Si_. *D*_H_ = 0.70(12) observed in this study will be independent from Si concentration in liquid Fe; the little interaction between Si and H in Fe is suggested from the fact that Si atoms substitute Fe, while H atoms occupy the interstitial sites^53^.

### Possible range of the outer core composition

The liquidus phase relations, in particular the liquidus field of Fe in the Fe–Si–H ternary system at the ICB pressure constrain the liquid core composition. Those determined at ~ 50 GPa (Fig. 3a) may be extrapolated to higher pressures based on the pressure evolutions of the Fe–FeSi and Fe–FeH binary eutectic liquid compositions (Fig. 3b). Si concentration in the Fe–FeSi eutectic liquid has been shown to decrease from 11.5 wt% at 50 GPa to 8 wt% at 330 GPa^22^. On the other hand, the Fe–FeH eutectic composition (Fe + 0.8 wt% H) at ~ 50 GPa would remain similar at higher pressures because the temperature/pressure slope of the melting curve of stoichiometric FeH is comparable to that of Fe at > 40 GPa^54^. The outer core composition should be within the liquidus field of Fe—the (Si, H)-depleted hcp phase—at 330 GPa (blue area in Fig. 3b) to form the dense inner core when both Si and H are important impurity elements. It is noted that the presence of other light elements such as O and S diminishes the Si and H variations in the liquidus field of Fe.

The possible range of the inner core composition proposed by Ref.^17^ for *T*_ICB_ = 6000–6500 K is Fe_60_Si_4_H_1_–Fe_60_Si_4_H_8_. If *T*_ICB_ = 5500 K, it can be Fe_60_Si_4_H_15_. With *D*_Si_ and *D*_H_, the compositions of liquids in equilibrium with these possible inner core solids are calculated to be Fe + 3.5(2) wt% Si + 0.04(1)–0.61(11) wt% H. Such liquid compositions are almost fully within the liquidus field of Fe at 330 GPa (Fig. 3b), which ensures that they crystallize hcp Fe–Si–H under inner core conditions. However, both *D*_Si_ = 0.94(4) and *D*_H_ = 0.70(12) are close to 1.0 and do not make large differences in Si and H concentrations between the outer and inner core. Indeed, such Fe–Si–H liquid compositions require additional light elements to explain the observed outer core density and velocity according to the ab initio calculations by Ref.^53^. Recent experiments on the metal-silicate partitioning of C during core formation suggested that the core contains at most 0.2 wt% C^55,56^. If O is an additional impurity element in the outer core (note that O is not soluble into the inner core and thus does not alter the possible range of the inner core composition considered here) and *T*_ICB_ = 6000 K, the outer core liquid may include 1.7–4.4 wt% O^53^ along with 3.5 wt% Si and 0.04–0.32 wt% H (Fe_60_Si_4.4–4.5_O_3.8–9.9_H_1.5–12_) (the O content can be lower when considering sulfur in the core). When *T*_ICB_ is 5500 K, we found liquid Fe + 3.5 wt% Si + 0.61 wt% H + 0.15 wt% O (Fe_60_Si_4.3_O_0.3_H_21_) for the outer core.

While the inner core temperature is still uncertain^2^, these results suggest that H and O, in addition to Si, are likely to be important light elements in the core (note that the liquid core constitutes 95% of the bulk core by mass). It supports recent arguments^3–5^ on the delivery of a large amount of water to the accreting Earth and its sequestration in metals during core formation for the most part^6–9^.

## Conclusions

Recent theoretical calculations^17^ found that hcp Fe_60_Si_4_H_1–15_ alloys, depending on core temperatures, account for the density and velocities observed in the Earth’s solid inner core. Our experiments demonstrated that liquid metal crystallized hcp Fe_60_Si_4.5_H_16_ which is close in composition to the predicted inner core alloys, indicating that hcp Fe can simultaneously include both Si and H unlike fcc FeH that does not incorporate Si. We determined the liquidus phase relations in the Fe–Si–H ternary system, suggesting the Fe–FeH eutectic composition to be around FeH_0.45_. We also obtained the solid hcp-Fe/liquid partition coefficients for Si and H, *D*_Si_ = 0.94(4) and *D*_H_ = 0.70(12). While these experiments were carried out at ~ 50 GPa, the *D*_H_ value is likely not sensitive to pressure because the size of H atom is substantially smaller than those of Fe and Si atoms even at 330 GPa^53^. Similar temperature/pressure slopes between the melting curves of Fe and stoichiometric FeH^54^ suggest that the Fe–FeH liquidus phase relations change little with increasing pressure. Therefore, the Fe–Si–H ternary liquidus diagram may be extrapolated to ICB conditions by primarily considering the change in the Fe–FeSi liquidus phase relations reported in previous studies^19,22^.

Such liquidus phase relations, in particular the liquidus field of Fe along with *D*_Si_ and *D*_H_ between the solid and liquid cores help constrain the Earth’s core composition. We explored the possible compositional range of the outer core liquid that is in equilibrium with the predicted solid inner core alloy^17^. The relatively high *D*_Si_ and *D*_H_ close to 1.0 do not make much differences in Si and H concentrations between the outer and inner core, requiring other light elements such as O that is least partitioned into solid Fe^10–12^. Depending on the ICB temperature that is still uncertain, we found H and O, as well as Si, are important core light elements, which support recent arguments on the sequestration of a large amount of water in the Earth’s core^6–9^.

## Supplementary Information

Supplementary Figures.

## Data Availability

All data supporting the findings of this study are available in the paper or from the corresponding author upon request.

## References

[1] Birch F (1952). Elasticity and constitution of the Earth’s interior. J. Geophys. Res..

[2] Hirose K, Wood B, Vočadlo L (2021). Light elements in the Earth’s core. Nat. Rev. Earth Environ..

[3] Raymond SN, Quinn T, Lunine JI (2007). High-resolution simulations of the final assembly of Earth-like planets. 2. Water delivery and planetary habitability. Astrobiology.

[4] Walsh KJ, Morbidelli A, Raymond SN, O'Brien DP, Mandell AM (2011). A low mass for Mars from Jupiter’s early gas-driven migration. Nature.

[5] Sato T, Okuzumi S, Ida S (2016). On the water delivery to terrestrial embryos by ice pebble accretion. Astron. Astrophys..

[6] Okuchi T (1997). Hydrogen partitioning into molten iron at high pressure: Implications for Earth’s core. Science.

[7] Li Y, Vočadlo L, Sun T, Brodholt JP (2020). The Earth’s core as a reservoir of water. Nat. Geosci..

[8] Yuan L, Steinle-Neumann G (2020). Strong sequestration of hydrogen into the Earth’s core during planetary differentiation. Geophys. Res. Lett..

[9] Tagawa S (2021). Experimental evidence for hydrogen incorporation into Earth’s core. Nat. Commun..

[10] Alfè D, Gillan MJ, Price GD (2002). Composition and temperature of the Earth’s core constrained by combining ab initio calculations and seismic data. Earth Planet. Sci. Lett..

[11] Ozawa H, Hirose K, Tateno S, Sata N, Ohishi Y (2010). Phase transition boundary between B1 and B8 structures of FeO up to 210 GPa. Phys. Earth Planet. Inter..

[12] Yokoo S, Hirose K, Sinmyo R, Tagawa S (2019). Melting experiments on liquidus phase relations in the Fe–S–O ternary system under core pressures. Geophys. Res. Lett..

[13] Mao Z (2012). Sound velocities of Fe and Fe–Si alloy in the Earth’s core. Proc. Natl. Acad. Sci. USA.

[14] Antonangeli D (2018). Sound velocities and density measurements of solid hcp-Fe and hcp-Fe–Si (9 wt.%) alloy at high pressure: Constraints on the Si abundance in the Earth’s inner core. Earth Planet. Sci. Lett..

[15] Shibazaki Y (2012). Sound velocity measurements in dhcp-FeH up to 70 GPa with inelastic X-ray scattering: Implications for the composition of the Earth’s core. Earth Planet. Sci. Lett..

[16] Sakamaki T (2016). Constraints on Earth’s inner core composition inferred from measurements of the sound velocity of hcp-iron in extreme conditions. Sci. Adv..

[17] Wang W, Li Y, Brodholt JP, Vočadlo L, Walter MJ, Wu Z (2021). Strong shear softening induced by superionic hydrogen in Earth’s inner core. Earth Planet. Sci. Lett..

[18] Kuwayama Y, Hirose K (2004). Phase relations in the system Fe–FeSi at 21 GPa. Am. Mineral..

[19] Fischer RA (2013). Phase relations in the Fe–FeSi system at high pressures and temperatures. Earth Planet. Sci. Lett..

[20] Tateno S, Kuwayama Y, Hirose K, Ohishi Y (2015). The structure of Fe–Si alloy in Earth’s inner core. Earth Planet. Sci. Lett..

[21] Ozawa H, Hirose K, Yonemitsu K, Ohishi Y (2016). High-pressure melting experiments on Fe–Si alloys and implications for silicon as a light element in the core. Earth Planet. Sci. Lett..

[22] Hasegawa M, Hirose K, Oka K, Ohishi Y (2021). Liquidus phase relations and solid–liquid partitioning in the Fe–Si–C system under core pressures. Geophys. Res. Lett..

[23] Fukai Y, Syono Y, Manghnani MH (1992). Some properties of the Fe–H system at high pressures and temperatures, and their implications for the Earth’s core. High-Pressure Research: Applications to Earth and Planetary Sciences.

[24] Sakamaki K (2009). Melting phase relation of FeHx up to 20 GPa: Implication for the temperature of the Earth’s core. Phys. Earth Planet. Inter..

[25] Shibazaki Y (2014). High-pressure and high-temperature phase diagram for Fe_0.9_Ni_0.1_-H alloy. Phys. Earth Planet. Int..

[26] Iizuka-Oku R (2017). Hydrogenation of iron in the early stage of Earth’s evolution. Nat. Commun..

[27] Machida A (2014). Site occupancy of interstitial deuterium atoms in face-centred cubic iron. Nat. Commun..

[28] Ikuta D (2019). Interstitial hydrogen atoms in face-centered cubic iron in the Earth’s core. Sci. Rep..

[29] Tagawa S, Ohta K, Hirose K, Kato C, Ohishi Y (2016). Compression of Fe–Si–H alloys to core pressures. Geophys. Res. Lett..

[30] Chi Z (2011). Cryogenic implementation of charging diamond anvil cells with H_2_ and D_2_. Rev. Sci. Instrum..

[31] Ohta K (2015). Phase boundary of hot dense fluid hydrogen. Sci. Rep..

[32] Antonov VE (2019). Solubility of deuterium and hydrogen in fcc iron at high pressures and temperatures. Phys. Rev. Mater..

[33] Mori Y (2017). Melting experiments on Fe–Fe_3_S system to 254 GPa. Earth Planet. Sci. Lett..

[34] Seto Y, Nishio-Hamane D, Nagai T, Sata N (2010). Development of a software suite on X-ray diffraction experiments. Rev. High Press. Sci. Technol..

[35] Dorogokupets PI, Dewaele A (2007). Equations of state of MgO, Au, Pt, NaCl-B1, and NaCl-B2: internally consistent high-temperature pressure scales. High Press. Res..

[36] Campbell AJ (2009). High pressure effects on the iron-iron oxide and nickel-nickel oxide oxygen fugacity buffers. Earth Planet. Sci. Lett..

[37] Akahama Y, Kawamura H (2004). High-pressure Raman spectroscopy of diamond anvils to 250 GPa: Method for pressure determination in the multimegabar pressure range. J. Appl. Phys..

[38] Hirose K (2017). Crystallization of silicon dioxide and compositional evolution of the Earth’s core. Nature.

[39] Fukai Y, Suzuki T (1986). Iron-water reaction under high pressure and its implication in the evolution of the Earth. J. Geophys. Res..

[40] Caracas R (2015). The influence of hydrogen on the seismic properties of solid iron. Geophys. Res. Lett..

[41] Dewaele A (2006). Quasihydrostatic equation of state of iron above 2 Mbar. Phys. Rev. Lett..

[42] Tsujino N (2013). Equation of state of γ-Fe: Reference density for planetary cores. Earth Planet. Sci. Lett..

[43] Edmund E (2019). Velocity-density systematics of Fe–5 wt%Si: Constraints on Si content in the Earth’s inner core. J. Geophys. Res. Solid Earth.

[44] Hirose K (2019). Hydrogen limits carbon in liquid iron. Geophys. Res. Lett..

[45] Edmund E (2019). Structure and elasticity of cubic Fe–Si alloys at high pressures. Phys. Rev. B.

[46] Fu S, Chariton S, Prakapenka VB, Chizmeshya A, Shim S-H (2022). Hydrogen solubility in FeSi alloy phases at high pressures and temperatures. Am. Mineral..

[47] Fei Y, Murphy C, Shibazaki Y, Shahar A, Huang H (2016). Thermal equation of state of hcp-iron: Constraint on the density deficit of Earth’s solid inner core. Geophys. Res. Lett..

[48] Tateno S (2018). Melting experiments on Fe–Si–S alloys to core pressures: Silicon in the core?. Am. Mineral..

[49] Tao R, Fei Y (2021). High-pressure experimental constraints of partitioning behavior of Si and S at the Mercury’s inner core boundary. Earth Planet. Sci. Lett..

[50] Yokoo S, Hirose K, Tagawa S, Morard G, Ohishi Y (2022). Stratification in planetary cores by liquid immiscibility in Fe–S–H. Nat. Commun..

[51] Li Y, Vočadlo L, Brodholt JP (2018). The elastic properties of hcp-Fe alloys under the conditions of the Earth’s inner core. Earth Planet. Sci. Lett..

[52] Badro, J., Côté, A. S. & Brodholt, J. P. A seismologically consistent compositional model of Earth’s core. *Proc. Natl Acad. Sci. USA* **111**, 7542–7545 (2014). 10.1073/pnas.1316708111 PMC4040578 24821817

[53] Umemoto K, Hirose K (2020). Chemical compositions of the outer core examined by first principles calculations. Earth Planet. Sci. Lett..

[54] Tagawa S, Helffrich G, Hirose K, Ohishi Y (2022). High-pressure melting curve of FeH: Implications for eutectic melting between Fe and non-magnetic FeH. Earth Space Sci. Open Arch..

[55] Fischer R, Cottrell E, Hauri E, Lee KKM, Le Voyer M (2020). The carbon content of Earth and its core. Proc. Natl. Acad. Sci. USA.

[56] Blanchard I (2022). The metal–silicate partitioning of carbon during Earth’s accretion and its distribution in the early solar system. Earth Planet. Sci. Lett..

[57] Tagawa S, Gomi H, Hirose K, Ohishi Y (2022). High-temperature equation of state of FeH: Implications for hydrogen in Earth’s inner core. Geophys. Res. Lett..

