# Supplementary material for: Melting phase relations in Fe–Si–H at high pressure and implications for Earth’s inner core crystallization
Source: Sci Rep. 2022 Jun 15;12:10000. doi: 10.1038/s41598-022-14106-z (PMC9200858; doi:10.1038/s41598-022-14106-z)
Supplement: Supplementary file 1 — Supplementary Figures. [file 41598_2022_14106_MOESM1_ESM.pdf]

Supplementary Information for

Melting phase relations in Fe-Si-H at high pressure and implications for Earth's inner core crystallization

Koutaro Hikosaka[1], Shoh Tagawa[1,2], Kei Hirose[1,2,*], Yoshiyuki Okuda[1], Kenta Oka[1], Koichiro Umemoto[2] & Yasuo Ohishi[3]

[1]Department of Earth and Planetary Science, The University of Tokyo, Bunkyo, Tokyo 113-0033, Japan.
[2]Earth-Life Science Institute, Tokyo Institute of Technology, Meguro, Tokyo 152-8550, Japan.
[3]Japan Synchrotron Radiation Research Institute, SPring-8, Sayo, Hyogo 679-5198, Japan.

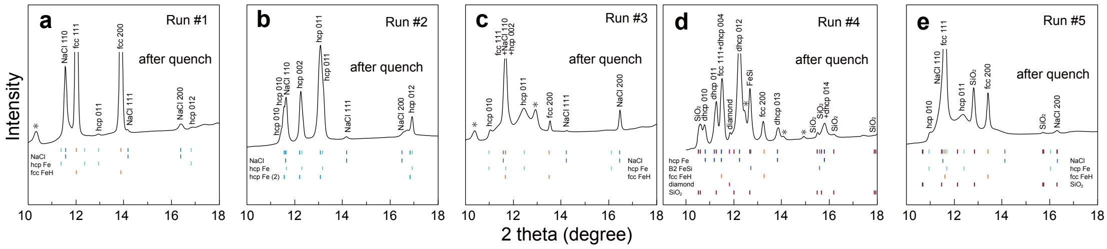

Figure S1. Sample XRD patterns collected after heating at high pressures and 300 K in (**a**–**e**) runs #1 to #5. Calculated peak positions for each phase are shown. Asterisks indicate unknown peaks.

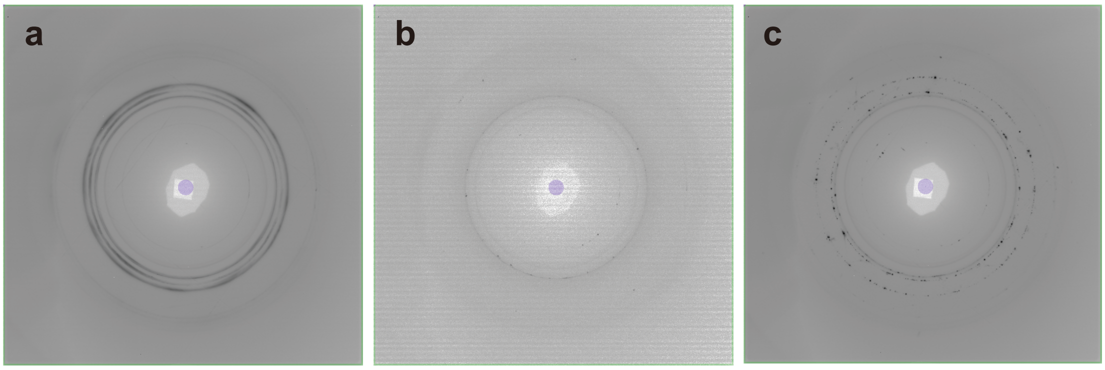

Figure S2. Two-dimensional XRD images collected before (**a**), during (**b**) and after heating (**c**) in run #1.

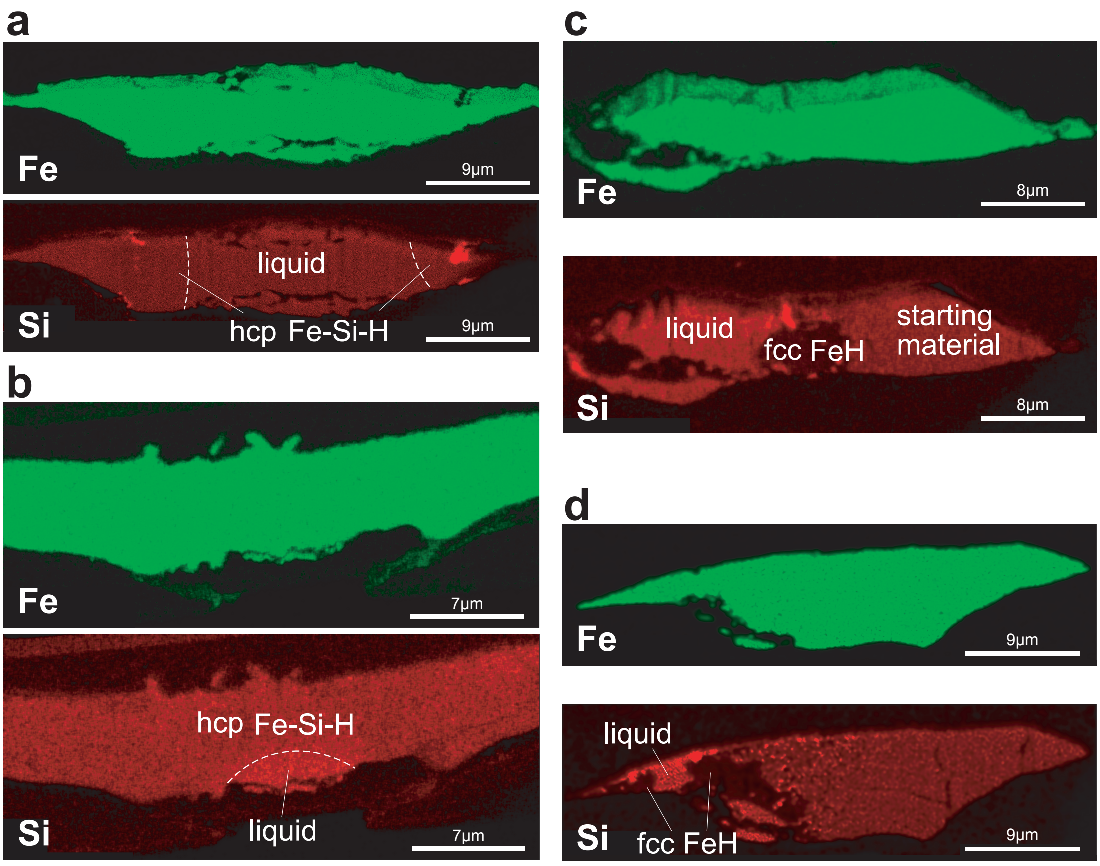

Figure S3. X-ray elemental maps of Fe (green) and Si (red) for samples recovered from runs #1 (**a**), #2 (**b**), #3 (**c**) and #5 (**d**). Liquid and coexisting solid phase are labelled. The liquid-solid boundary was drawn based on the compositional difference or the intrusion of NaCl into the alloy part. See Fig. 2 for run #4.

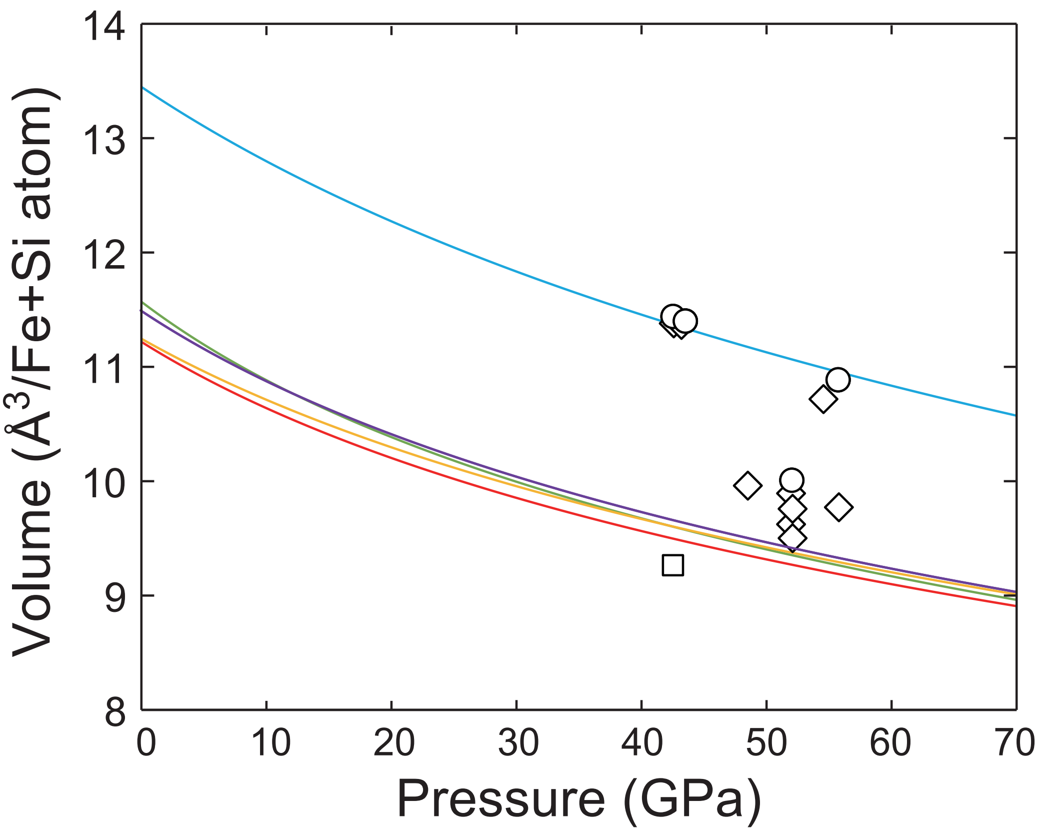

Figure S4. The volumes of the hydrogen-bearing hcp/dhcp (diamonds), fcc (circles), and B2 phases (square) found in this study. They are compared to the compression curves of hydrogen-free hcp Fe (red[41]), hcp Fe+6.5wt%Si (yellow[20]), fcc Fe (green[42]), fcc FeH (blue[57]) and B2 Fe+12.0wt%Si (purple[45]).
